# A Real Pandora’s Box in Pandemic Times: A Narrative Review on the Acute Cardiac Injury Due to COVID-19

**DOI:** 10.3390/life12071085

**Published:** 2022-07-20

**Authors:** Amalia-Stefana Timpau, Radu-Stefan Miftode, Daniela Leca, Razvan Timpau, Ionela-Larisa Miftode, Antoniu Octavian Petris, Irina Iuliana Costache, Ovidiu Mitu, Ana Nicolae, Alexandru Oancea, Alexandru Jigoranu, Cristina Gabriela Tuchilus, Egidia-Gabriela Miftode

**Affiliations:** 1 Department of Infectious Diseases (Internal Medicine II), Faculty of Medicine, University of Medicine and Pharmacy “Gr. T. Popa”, 700115 Iasi, Romania; amalia-stefana-v-darie@d.umfiasi.ro (A.-S.T.); lecadaniela@ymail.com (D.L.); ionela-larisa.miftode@umfiasi.ro (I.-L.M.); egidia.miftode@umfiasi.ro (E.-G.M.); 2 Department of Internal Medicine I (Cardiology), Faculty of Medicine, University of Medicine and Pharmacy “Gr. T. Popa”, 700115 Iasi, Romania; antoniu.petris@umfiasi.ro (A.O.P.); ovidiu.mitu@umfiasi.ro (O.M.); nicolaeana2001@yahoo.com (A.N.); oancea.alexandru-florinel@email.umfiasi.ro (A.O.); jigoranu.raul-alexandru@email.umfiasi.ro (A.J.); 3 Department of Radiology and Medical Imaging, St. Spiridon Emergency Hospital, 700115 Iasi, Romania; razvan-timpau@email.umfiasi.ro; 4 Department of Preventive Medicine and Interdisciplinarity (Microbiology), Faculty of Medicine, University of Medicine and Pharmacy “Gr. T. Popa”, 700115 Iasi, Romania; cristina.tuchilus@umfiasi.ro

**Keywords:** myocardial injury, COVID-19, cytokines, biomarkers, heart failure

## Abstract

The intricate relationship between severe acute respiratory syndrome coronavirus 2 (SARS-CoV-2) and the cardiovascular system is an extensively studied pandemic topic, as there is an ever-increasing amount of evidence that reports a high prevalence of acute cardiac injury in the context of viral infection. In patients with Coronavirus disease 2019, COVID-19, a significant increase in serum levels of cardiac troponin or other various biomarkers was observed, suggesting acute cardiac injury, thus predicting both a severe course of the disease and a poor outcome. Pathogenesis of acute cardiac injury is not yet completely elucidated, though several mechanisms are allegedly involved, such as a direct cardiomyocyte injury, oxygen supply-demand inequity caused by hypoxia, several active myocardial depressant factors during sepsis, and endothelial dysfunction due to the hyperinflammatory status. Moreover, the increased levels of plasma cytokines and catecholamines and a significantly enhanced prothrombotic environment may lead to the destabilization and rupture of atheroma plaques, subsequently triggering an acute coronary syndrome. In the present review, we focus on describing the epidemiology, pathogenesis, and role of biomarkers in the diagnosis and prognosis of patients with acute cardiac injury in the setting of the COVID-19 pandemic. We also explore some novel therapeutic strategies involving immunomodulatory therapy, as well as their role in preventing a severe form of the disease, with both the short-term outcome and the long-term cardiovascular sequelae being equally important in patients with SARS-CoV-2 induced acute cardiac injury.

## 1. Introduction

The deadliest pandemic in the modern era is currently ongoing and is caused by severe acute respiratory syndrome-coronavirus-2 (SARS-CoV-2) which is the most recent member of the coronavirus family to emerge this century, after the severe acute respiratory syndrome coronavirus (SARS-CoV) and the Middle East respiratory syndrome coronavirus (MERS-CoV) in the early 2000s [1]. Most respiratory infections usually evolve with mild to moderate symptoms, but coronaviruses drew attention that viral pneumonia may also progress to severe forms further complicated with multiple organ failure [2,3]. Several studies analyzed the short- and mid-term impact of cardiovascular complications, highlighting that acute cardiac injury in patients with a confirmed diagnosis of Coronavirus disease 2019 (COVID-19) represents significant associated morbidity [4,5,6,7]. Moreover, additional research investigating the long-term sequelae of COVID-19 indicates a substantial risk of cardiovascular complications, particularly heart failure (HF) and atrial fibrillation, even in patients not requiring hospitalization during the acute phase of the viral infection [8].

Diagnosis of acute cardiac injury is outlined using the cardiac troponin (cTn) assay above the 99th percentile as the upper reference limit. Myocardial injury is considered acute if the dynamic cTn concentration exceeds the biological variation limits [9]. However, one should consider that a personal history of cardiovascular disease and elevated levels of cTn are factors of poor prognosis, being significantly associated with an increased risk of mortality in patients hospitalized with COVID-19. Specifically, two meta-analyses reported an 8 to 21-fold higher risk of fatal events in patients with SARS-CoV-2-related acute cardiac injury [10,11]. A plethora of studies show that approximately one in every three patients with COVID-19 will develop a certain phenotype of myocardial injury [7,12,13], the risk being significantly higher in patients with previously diagnosed chronic coronary heart disease [14]. Additional supporting evidence concerning acute cardiac injury due to coronavirus infection was provided by a large meta-analysis performed by Abate et al. which reported an alarming 22.3% prevalence of myocardial injury in patients who tested positive for SARS-CoV-2 [10].

Despite the increased risk of myocardial infarction associated with COVID-19, starting with the onset of the pandemic, various literature data paradoxically reported an up to 50% reduction in hospitalization rates due to this cardiovascular pathology. This might be either a consequence of patients unwilling to risk exposure to a SARS-CoV-2 infection or due to the significant reduction in available hospital beds for “non-COVID-19” patients [15,16]. Furthermore, a restraint for percutaneous coronary intervention (PCI) procedures was observed particularly in patients with a confirmed or just even suspected COVID-19 diagnosis. Non-infected patients received primary PCI as a conventional approach, while in patients positive for SARS-CoV-2, the strategy focused on fibrinolysis in a higher number of cases, as the latter represents a therapeutic option that involves a lower interaction and, subsequently, lower exposure to viral particles [17,18]. 

The possible occurrence of cardiovascular complications in SARS-CoV-2 infection, the risk factors and specific symptoms of myocardial injury, and the implementation of adequate investigations and treatment have a profound impact on the patients’ prognosis [19]. There is still no management consensus for patients with acute cardiac injury; the constant pandemic-related challenge for clinicians is to continuously balance the benefits of a comprehensive cardiac evaluation and the risk of spreading the infection. Supportive care and an integrative approach to the underlying infectious disease may represent a feasible standard of care in patients with COVID-19-associated acute cardiac injury [20].

In this regard, the present review is conceived to provide an overview on SARS-CoV-2 induced acute cardiac injury, describing the allegedly involved mechanisms, highlighting the contribution of cardiac biomarkers in both the initial diagnostic approach and subsequent follow-up of COVID-19-related acute cardiac injury, as well as pointing out some state-of-the-art data for therapeutic strategies to address it.

## 2. Cardiovascular Pathophysiology Related to COVID-19 and the Most Common Clinical Phenotypes

### 2.1. The Pathophysiological Continuum between Infection and Cardiac Injury

Coronaviruses have a specific crown-like surface appearance and a structure made from four structural proteins, known as the spike (S), envelope (E), membrane (M), and nucleocapsid (N) proteins. SARS-CoV-2 enters the cell after the proteolytic cleavage of the S protein followed by its binding to the cell-surface receptor angiotensin 2 conversion enzyme (ACE 2) [21]. The latter is a membrane protein that plays a vital role in the cardiovascular and immune systems and is well expressed in the lung and heart endothelial cells, macrophages, and cardiomyocytes [22]. ACE 2 levels are higher in patients receiving treatment with medications that inhibit the renin-angiotensin-aldosterone system, such as ACE inhibitors or angiotensin receptor blockers [23]. The use of these neurohormonal modulating drugs was controversial in the early stages of the pandemic. Nevertheless, multiple studies conducted during the last two years have demonstrated that is not advisable to interrupt their chronic administration, nor to change the therapeutic class. Basically, the current consensus emphasizes that maintaining the previously prescribed treatment does not adversely affect the course of viral infection and continues to provide cardiac protection at the same time [24,25,26]. Moreover, ACE 2 has been shown to provide additional protective effects against pulmonary injury in patients with ARDS due to severe forms of COVID-19 [27].

A SARS-CoV-2 infection may both exacerbate pre-existing cardiovascular comorbidities and trigger new ones. HF is among the most frequently reported cardiovascular complications related to COVID-19 infection, being diagnosed in up to 24% of patients, while venous thromboembolism was identified in 21% of cases, followed by dysrhythmias and myocarditis in 17% and 7% of patients, respectively [5,6,27,28]. The main risk factors for SARS-CoV-2-induced acute cardiac injury include smoking, male gender, and comorbidities, including diabetes mellitus, arterial hypertension, and coronary artery disease [10,11]. The imbalance between pro-inflammatory and anti-inflammatory mediators may be responsible for the development of major cardiovascular events [29]. The response to infection is governed by the adequate activity of both the innate and acquired immune systems. 

Two major immunity defects are required for the onset of the critical illness in COVID-19 which is frequently associated with myocardial injury. The first is represented by a lack of initial control over the viral clearance, mainly through the innate immune system, while the second resides in the inability to regulate a balanced production of pro- and anti-inflammatory cytokines [2,30]. The innate immune response to viral infection is based on interferon types I and III. On one hand, in SARS-CoV-2 infection, interferon levels were observed to increase only in critical patients, whereas in all other positive cases the immune response was reduced and/or delayed. On the other hand, the response was quicker and more vigorous in patients hospitalized for influenza pneumonia. This particular ability of SARS-CoV-2 to elude the early innate immune response may lead to an insufficient viral clearance, followed by the development of a hyperinflammatory condition, and, in certain categories of patients, the onset of acute respiratory distress syndrome (ARDS) [31,32].

Moreover, the innate immunity may also trigger the adaptive immunity consisting of CD4+ and CD8+ T cell lymphocytes and B lymphocytes that produce neutralizing antibodies. The combined T and B cell responses contribute to SARS-CoV-2 infection resolution and a robust immunity [32]. Cytokines, predominantly produced by macrophages, mast cells, and dendritic cells, but also by B and T lymphocytes, play a central role in coordinating the immune response. A balanced synthesis of cytokines is required for an effective antiviral effect, but their excessive serum levels may cause a cytokine storm with massive collateral damage to vascular structures and alveolar barriers [33].

Cardiac troponin levels have been shown to be linearly correlated with C-reactive protein levels, indicating that acute cardiac injury may be closely related to systemic inflammation [34]. The distinctive pathogenesis of vascular damage is promoted by the presence of the cellular receptor of SARS-CoV-2 on the vascular cell’s surface, contributing to endothelial dysfunction [35]. Furthermore, direct infection of the endothelial lining may lead to vasculitis and apoptosis. As a result, the exposed subendothelial surface facilitates platelet aggregation in an attempt to repair the vascular damage. However, even in the absence of direct endothelial invasion, high levels of inflammatory cytokines have the ability to impair the endothelial function by increasing the number of adhesion molecules, thus promoting thrombogenic processes [36,37,38]. Coagulopathic events include thrombin generation, platelet consumption, and increased levels of fibrinogen and the Von Willebrand factor [39]. Viral coagulopathy is manifested by venous thrombosis, usually further complicated with venous thromboembolism but also by arterial thrombosis, most commonly found in myocardial or cerebral territories. Parenteral anticoagulants, such as unfractionated heparin and low molecular weight heparins, are routinely used in the treatment of patients admitted with moderate-to-severe COVID-19, while direct oral anticoagulants are predominantly used in the ambulatory management of patients presenting a mild form of the disease [40].

### 2.2. Acute Coronary Syndromes in the COVID-19 Pandemic: The “Perfect” Cardio-Inflammatory Symbiosis

The interplay between atherosclerotic disease and inflammation has been well-described, starting with the formation of atheroma plaque up to its erosion and rupture, with the subsequent occurrence of an acute coronary syndrome [41]. Total occlusion of a coronary artery induced by an eroded, vulnerable atherosclerotic plaque and the overlying thrombus formation might be a consequence of a hyperinflammatory environment along with the prothrombotic state induced by SARS-CoV-2 infection. This mechanism represents the basic pathophysiological substrate of ST-elevation myocardial infarction (STEMI) but also accounts for approximately 25% of cases admitted with non-ST-elevation myocardial infarction (NSTEMI) [42,43]. The real epidemiological impact of COVID-19 in patients with cardiac ischemia is mirrored by the worrisome incidence rates of myocardial infarction among SARS-CoV-2 positive cases, ranging from 1.1% to 8.9% [44,45]. The risk of developing myocardial infarction is significantly higher in the early stages of the infection, with a 5-fold increase in risk during the first 14 days of COVID-19, compared to the pre-illness period [46].

Those high figures can be explained, at least partially, by certain similarities concerning the inflammatory pathways operating both in COVID-19 and atherosclerosis. Even before the COVID-19 pandemic, the American Heart Association (AHA) suggested that viral infections could destabilize the atherosclerotic plaques, and various collagenolytic enzymes, such as matrix metalloproteinases (MMP), were becoming associated with increased plaque vulnerability [47,48]. Those MMPs can be activated by a plethora of cytokines (e.g., TNF-alpha, INF-γ, IL-1, and IL-6), thus diminishing the cohesion of the atherosclerotic plaque and consecutively increasing the risk of acute coronary syndromes [48,49]. Based on these observations, the COVID-19 pandemic again turned the spotlight on statins’ pleiotropic effects, primarily based on their anti-inflammatory response, doubled by the amelioration of the endothelial function. The plaque stabilization occurs via the enhanced calcification and thickening of the fibrous cap, with inflammation playing a central role. The large JUPITER study even highlighted that patients treated with rosuvastatin presented decreased serum levels of C-reactive protein and a reduced apparition of major cardiovascular events, compared to the placebo group [50,51], while a large Swedish study claimed that previous chronic treatment with statins exhibited a modest preventive therapeutic effect on COVID-19 mortality [52]. The continuation and/or initiation of statins in COVID-19 patients may also be beneficial from the perspective of the lipid profile, as hypercholesterolemia is associated with an increased susceptibility to SARS-CoV-2 infection. It was demonstrated that high cholesterol levels are associated not only with increased density of ACE2 receptors on host cell membranes but also with a more effective interaction between the viral spike protein and the ACE2 receptors [53].

In addition, significant platelet activation occurs during the systemic inflammation associated with SARS-CoV-2 infection. This phenomenon is induced by the binding of pro-inflammatory interleukins to the platelet surface receptors, and by reducing the availability of endothelial nitric oxide. At the same time, neutrophils express adhesion molecules favoring platelet aggregation. These mechanisms, together with endothelial injury, facilitate the interaction between the platelets and the endothelial cells, thus aggravating the thromboinflammatory pathways, representing a hallmark for COVID-19 [54].

Even if mortality caused by acute myocardial infarction has reached its lowest level in the era of PCI, it still remains associated with considerable morbidity. Restoring adequate myocardial reperfusion in a timely manner limits the area of the infarction and significantly improves the outcome, regardless of the associated pathologies [55]. However, contradictorily, the resumption of blood flow to the ischemic area may lead to additional myocardial damage, a phenomenon known as reperfusion myocardial injury. This paradoxical mechanism may be responsible for the loss of up to 50% of viable myocardium and elevated cytokine levels, in addition, biochemical and metabolic changes caused by hypoxia play a core role in its occurrence [56]. A cohort study including patients over the age of 65 showed that more than three-quarters (76%) of patients who survived a first acute myocardial infarction developed HF in the next 5 years [57]. Despite modern reperfusion strategies and neurohormonal blocking therapies, the incidence of HF remains unacceptably high and there is an urgent need for better management in order to improve both survival and quality of life after myocardial infarction. However, abnormally activated immune responses during infection with SARS-CoV-2 lead to a suboptimal myocardial repair with a higher incidence of HF [58].

Type 2 myocardial infarction is caused by the imbalance between a deficient myocardial oxygen supply and an increased metabolic demand, due to specific cardiac and non-cardiac pathological conditions.

A variable association of some commonly met mechanisms in COVID-19 patients seems relevant in this context:

(1) Previously stable coronary artery disease that limits myocardial perfusion;
(2) Endothelial dysfunction in the coronary microcirculation;
(3) Significantly increased arterial hypertension resulting from elevated circulating levels of Angiotensin II and catecholamines;
(4) Hypoxemia due to acute respiratory distress syndrome (ARDS) or in situ pulmonary vascular thrombosis. In the case of sepsis, pulmonary injury, and respiratory failure, significant increases in biomarkers of overload and myocardial injury can be noticed [12,59].

Moreover, infections in general, and COVID-19-associated pneumonia in particular, can unbalance the thin equilibrium between myocardial O_2_ supply and consumption. The increase in the physiological demand for O_2_ caused by systemic infection can be so significant that this imbalance occurs even in the absence of angiography-relevant atherosclerotic plaques. Several studies emphasized this pathway as the main mechanism of COVID-related acute cardiac injury. Essentially, vasodilation represents the main pathophysiological mechanism of the response of the cardiovascular system to sepsis. In addition, hypotension is the natural consequence of vasodilation, which can even progress to hemodynamic collapse, thus inducing or aggravating coronary hypoperfusion with subsequent acute myocardial injury through a reduced O_2_ supply [60]. At the same time, in the context of sepsis, reflex tachycardia increases the myocardial oxygen demand. Of course, the presence of atheroma plaques is a risk factor for the unfavorable evolution in patients with sepsis, increasing the risk of acute myocardial injury [61,62], but COVID-19 is also highly associated with non-atherosclerotic coronary perfusion impairment, such as spasm of the coronary arteries, dissection of the coronary wall, microthrombosis in the context of the hypercoagulant state, or vasculitis-like injury of the coronary vessels [63,64]. Regardless of the intimate mechanism of COVID-19-related myocardial infarction with non-obstructive coronary arteries, the patients’ prognosis is poor, with high mortality rates mainly due to the increased prevalence of severe comorbidities, such as ARDS, obesity, or congenital thrombophilia [64,65].

Under these circumstances, it is difficult to clearly differentiate between patients with acute coronary syndromes, such as unstable angina or NSTEMI, and those with acute myocarditis or myocardial injury caused exclusively by metabolic imbalances in the context of fever, tachycardia, or hypoxemia due to ARDS [59]. Those patients require an integrative diagnostic and therapeutic approach, focusing not only on SARS-CoV-2 infection and the major associated cardiovascular pathology but also on the frequently coexisting factors of poor prognosis.

### 2.3. Heart Failure in COVID-19 Patients: Different Pathways, Same Target

Patients admitted for COVID-19 may develop either an acute decompensation of a chronic, previously stable HF or a de-novo acute HF as an immediate consequence of acute cardiac injury [66]. Pathogenesis of COVID-19 cardiomyopathy is intimately related to inflammatory cytokines, referring here to diastolic dysfunction and increased myocardial stiffness mediated by interleukin-6 (IL-6), negative inotropic effects exerted by interleukin-1β (IL-1β), or myocardial fibrosis induced by IL-1β and tumor necrosis factor alfa (TNF-α). Even higher levels of those biomarkers are detected during cytokine storms in the severe forms of SARS-CoV-2 infection [67,68,69]. A significantly increased incidence of acute HF was reported in patients deceased due to severe COVID-19, as compared to their survivor counterparts [70]. Moreover, the in-hospital mortality rate in patients presenting both acute HF and COVID-19 was extremely high, reaching up to 44.1% at the peak of the pandemic. Beyond the acute phase, COVID-19 may be responsible for HF as a long-term cardiovascular complication, but further clinical studies are required [66].

### 2.4. Myocarditis in COVID-19: Between Certainties and Controversies

The correlation between human coronaviruses and myocarditis is well-established [71]. Concerning SARS-CoV-2 infection, three pathophysiological mechanisms may contribute to myocarditis occurrence in patients with COVID-19. Firstly, it is worth mentioning the direct viral cardiomyocytes’ invasion with subsequent injury accompanied by various immune mechanisms such as T cell-mediated cytotoxicity and cytokines’ negative inotropic effects. Additionally, the autoimmune mechanisms triggered as a response to the release of cryptic antigens from cardiomyocytes following SARS-CoV-2-induced lesions could also enhance the development of myocarditis [72,73,74]. A very recent extensive study, including more than 100,000 subjects diagnosed with COVID-19, showed a 2 to 3-fold higher risk of myocarditis among infected patients [75], while the net prevalence of myocarditis among cases that required hospitalization was 2.4 per 1000 admissions [76]. Importantly, myocarditis was far more prevalent among non-vaccinated young males, compared to their non-vaccinated counterparts [77].

There is also evidence to support the hypothesis of molecular mimicry [78]. Necropsy studies that included endomyocardial biopsy suggest that direct viral toxicity is not the main mechanism of myocardial injury, as current evidence indicates that viral presence in the heart tissue is not necessarily associated with myocarditis [79]. Local myocardial inflammation, as well as severe systemic inflammation, can be a direct cause of myocardial injury in COVID-19 cases. It is already known that patients with sepsis-associated cardiomyopathy have an exacerbated inflammatory status that is characterized by elevated circulating levels of several cytokines, including the previously-mentioned IL-6 and TNF-α [59]. In vitro exposure to IL-6 has reduced the cardiomyocyte contractility, while recombinant TNF-α administration decreased the ejection fraction of the left ventricle in experimental models. Mechanisms of these myocytotoxic effects include the modulation of calcium channels’ flows and nitric oxide synthesis which are thought to play a major role in depressing myocardial function in sepsis [59,80,81]. The reversible acute cardiac dysfunction occurring in the context of a septic environment is known as sepsis-induced cardiomyopathy (SICM). Immune response to infection leads to mitochondrial dysfunction, disruption of contractile apparatus by altering calcium balance, and myocyte apoptosis [82,83]. Increased levels of cTn detected in SICM may also appear as a consequence of myocardial edema [84].

However, it remains unclear to what extent myocarditis is caused by direct viral myocardial damage or is just a consequence of systemic inflammation. 

### 2.5. Stress Cardiomyopathy: An Additional Trigger

The incidence of stress cardiomyopathy (also known as tako-tsubo cardiomyopathy) during the COVID-19 pandemic appears to follow an increasing trend, with psychological distress and anxiety having a core role in its onset. Incriminated mechanisms include the sympathetic activation causing catecholamine-induced myocardial stunning and microvascular dysfunction that is transient and more frequently observed in elderly women [85]. It is worth mentioning that a study showed that this hypercatholaminergic condition due to cytokine storm in critical patients (from ICU departments) induces a significantly increased blood pressure, compared to non-critical patients (145 mmHg vs. 122 mmHg; *p* < 0.001); interestingly, this hypertensive pattern in patients with severe forms of COVID-19 is actually associated with an improved prognosis, a lower need for inotropic support, and a decreased risk of developing cardiogenic shock or multiple organ dysfunction [86].

### 2.6. Right Ventricular Failure: A Key Element in the Hemodynamics of COVID-19 Patients

The right ventricle (RV) represents an essential component in the hemodynamic homeostasis of patients with COVID-19, an acute RV dysfunction being considered a factor of poor prognosis. Several mechanisms have a complementary role in RV injury [87,88]:

- Pulmonary hypertension induced by vasoactive mediators;
- Pulmonary vasoconstriction due to hypoxemia;
- Vascular remodeling;
- Microthrombi in pulmonary vessels due to inflammatory cytokines;
- Mechanical compression due to atelectasis, interstitial edema, or associated pleural effusion.

Moreover, in ICU-admitted patients due to a severe course of COVID-19, persistent ventilation with elevated positive expiratory pressures leads to a significantly increased RV afterload, thus inducing additional mechanical strain in a cavity with rather thin walls and further reducing an already impaired cardiac output [88]. This pathophysiological chain of events is clearly expressed by the rise of serum concentrations for several biomarkers, such as NT-proBNP, ST2, or GDF-15 molecules, that express various mechanisms suggestive not only of myocardial dysfunction but also of inflammation or oxidative stress, conditions that are commonly found in infected patients [89,90,91].

## 3. Biomarkers in the Diagnosis and Prognosis of Acute Myocardial Injury Due to COVID-19: Classic Approach, Novel Challenge

### 3.1. Cardiac Troponins 

**Figure 1:**
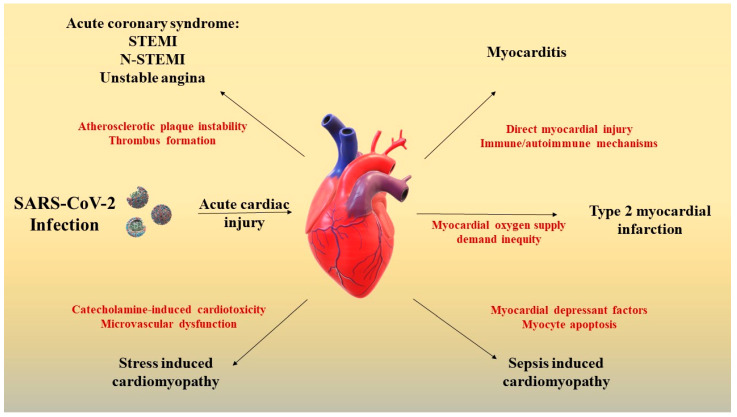
Potential pathophysiological mechanisms of SARS-CoV-2 induced acute cardiac injury; acute coronary syndromes occur by destabilization and rupture of atheroma plaques with the consequent formation of a thrombus that causes occlusion of the artery responsible for heart attack [41]. Myocarditis can occur through direct myocardial injury and immune or autoimmune mechanisms [72,73,74]. Type 2 myocardial infarction occurs due to an imbalance between the need and supply of oxygen [12]. Sepsis-induced cardiomyopathy occurs due to myocytic apoptosis and cardiodepressant factors [82,83]. Stress-induced cardiomyopathy occurs through the cardiotoxic effect of catecholamines and is secondary to microvascular dysfunction [85]. Legend: STEMI: ST-elevation myocardial infarction; and N-STEMI: non-ST-elevation myocardial infarction.

Despite the fact that diagnostic utility and performance of cTn in coronary heart disease are well known [92], the constantly reported high levels of cTn in COVID-19 patients triggered confusion among clinicians since the beginning of the pandemic [73,93,94,95,96]. A sudden cTn elevation may be suggestive of STEMI/N-STEMI, type 2 myocardial infarction, myocarditis, stress cardiomyopathy, sepsis-induced cardiomyopathy, or non-ischemic myocardial injury, each of these pathologies having different therapeutic approaches (Figure 1). A modest rise in cTn levels is seen in patients with mild and moderate forms of COVID-19, without influencing the clinical outcome [97]. The rise of cTn serum levels in patients who succumb to SARS-CoV-2 infection shows a particular pattern as compared to the concentration dynamics that characterize acute coronary syndromes (ACS). In COVID-19, there is a steady upward trend of cTn serum levels while ACS is generally observed as a very typical abrupt rise [98]. Nevertheless, a cTn elevation can also be detected in certain non-cardiac conditions, such as systemic inflammatory response syndrome, sepsis, pulmonary embolism, critical illness, or end-stage renal disease [99]. In fact, no overt ischemia is found in as many as 60% of cases where an acute cardiac injury is identified [12]. Therefore, a prompt differential diagnosis and a timely intervention in patients with SARS-CoV-2-related acute cardiac injury are mandatory [10].

In terms of prognosis, several meta-analyses reported that high serum levels of this biomarker are significantly correlated with increased mortality risk [10,98]. The ability of cTn concentration at admission to predict in-hospital mortality was outlined by an area under a curve greater than 0.90 (ROC analysis) [3,98,100]. In addition to being directly associated with major cardiovascular events, elevated cTn levels are also related to an increased risk of respiratory, hepatic, and renal impairment [101]. Serial measurements of cTn levels in patients with high initial values detected at admission are recommended either to confirm or to exclude myocardial infarction [102]. Taking into account the frequently elevated cTn serum levels, the question of whether it should be routinely assessed in COVID-19 pneumonia was raised. However, the American College of Cardiology stated in a recent review that clinicians are advised to measure cTn only in selected cases, either in patients with preexisting cardiovascular comorbidities or in clinical circumstances highly suggestive of ischemic etiology [103].

### 3.2. The Interleukins 

The interleukin-1 family comprises 11 cytokines with core roles in innate and adaptive immunity [104]. IL-1β represents the “apical” cytokine of the innate immune system, as it regulates cytokine and chemokine synthesis, and self-catalyzes its own production. In the acute inflammatory response phase to viral infection, an early increase in serum IL-1β and TNF-α levels are observed within the first 30 min, followed by a rise in IL-6 levels [105]. While IL-1β and TNF-α levels may drop within 24–48 h, elevated IL-6 levels persist for a longer period over the course of the infection [48]. Despite its short half-life, as an upstream cytokine, IL-1β may contribute to CRS development, inducing IL-6 production, leading to macrophage activation and pyroptosis, and caspase-1-dependent host cell death mediated by inflammatory cytokines [104,106,107,108]. The pathological consequence of pyroptosis is represented by endothelial cell inflammation with subsequent endothelial dysfunction and is potentially the initial step in the continuum of myocardial injury [39].

**Figure 2:**
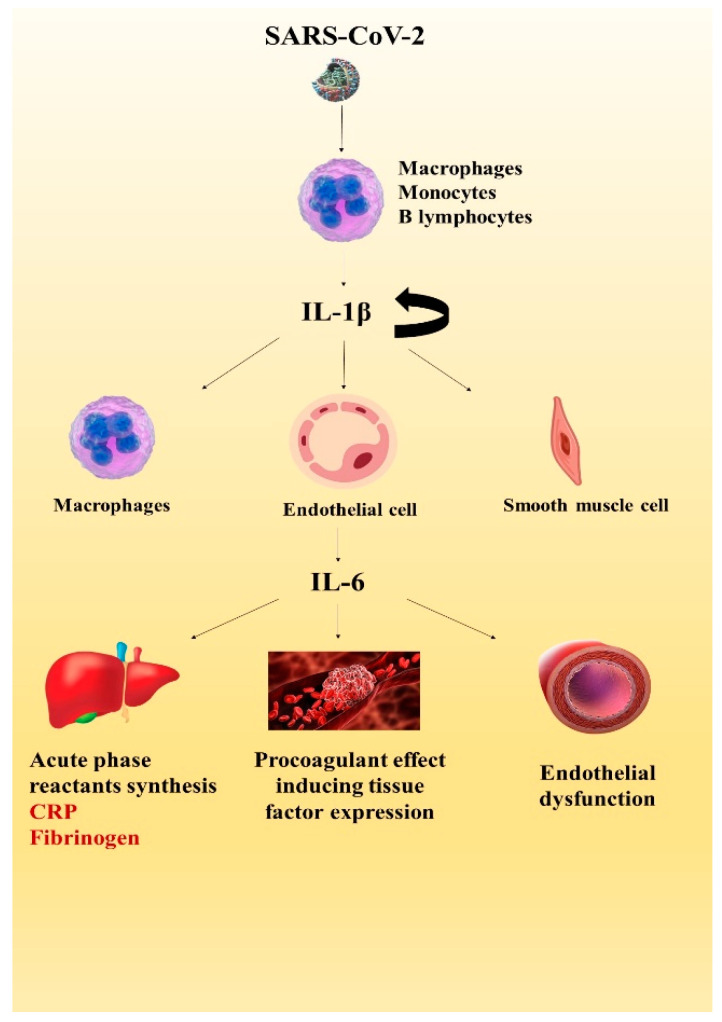
The inflammatory cascade during SARS-CoV-2 infection. IL-1β is synthesized by macrophages, monocytes, and B lymphocytes, and has the ability to self-induce its production and stimulate the synthesis of IL-6 in macrophages, endothelial cells, and smooth muscle cells [12,96,97]. Further, IL-6 determines the liver synthesis of acute phase reactants and exerts a pro-coagulant effect by inducing tissue factor synthesis, also causing endothelial dysfunction by increasing vascular permeability [108,109,110]. Legend: IL-1β: Interleukin 1β; IL-6: Interleukin-6; and CRP: C reactive protein.

In acute myocardial infarction, IL-1 plays multiple roles in the injury, repair, and remodeling processes, as depicted in Figure 2 [109]. Infarcted cardiomyocytes cause the release of IL-1α, epithelial injury stimulates the release of IL-1β, while monocytes are a source of IL-1β in acute coronary syndromes [110,111]. The inflammatory reaction that is normally involved in the clearance of dead cells and matrix detritus, could also extend the area of myocardial damage. Consequently, an auto-inflammatory loop is induced, as IL-1 recruits and activates more innate immune cells [43,111]. Stopping this inflammatory reaction is not a passive process, requiring anti-inflammatory mediators such as IL-10 and Transforming Growth Factor (TGF)-β which inactivate mononuclear cells and inhibit the transcription of IL-1, as well as other pro-resolving lipid mediators [43,109,112]. In ACS, elevated IL-1β levels are associated with diastolic dysfunction and remodeling [110,113]. Patients with more severe forms of COVID-19 might be at high risk, as it was demonstrated that elevated levels of IL-6 and high IL-1β levels are correlated with a poor prognosis in the setting of ACS [110,114].

Interleukin-6 family members, such as IL-6, IL-11, IL-30, IL-31, OSM, LIF, CNTF, CT-1, CT-2, and CLC, have an important role in heart disease pathogenesis as they exhibit pro-inflammatory, anti-inflammatory, and proatherogenic effects using different trans-signaling pathways [115]. IL-6 is synthesized by T lymphocytes, macrophages, and adipocytes, as well as by cardiovascular system structures such as endothelial cells, vascular smooth muscle cells, or ischemic cardiomyocytes [116]. It promotes inflammation through two signaling pathways, the *cis*-signaling pathway that uses the membrane IL-6 receptor and the *trans*-signaling pathway utilizing the soluble IL-6 receptor. The latter mechanism is utilized for activating and promoting inflammation in cells without the membrane receptor for IL-6 (e.g., endothelial cells) [117,118]. Increased IL-6 levels are essential for regulating the immune response, inducing the hepatic synthesis of acute phase biomarkers, and activating immune system cells and intercellular signaling. Through these mechanisms, IL-6 actively contributes to host defense, initiating the healing process of tissue injury [119]. By inducing the tissue factor expression on endothelial cells, this pro-inflammatory cytokine may also contribute to the occurrence of thrombotic complications [120].

IL-6 also plays a role in hematopoiesis, neurogenesis, and liver regeneration [121,122,123]. On one hand, it exhibits a short-term, prompt pro-inflammatory response for combating SARS-CoV-2 infection, thus having a protective effect [124]. On the other hand, the continuous synthesis of IL-6 has deleterious consequences, contributing to the development of inflammatory and autoimmune diseases [125]. The polymorphism of IL-6 is also observed at the cardiac level when its dual role is initially protective by limiting myocyte injury during acute myocardial infarction. In contrast, persistently elevated IL-6 levels in patients with acute myocardial infarction are associated with low left ventricular ejection fraction and HF [124,126].

IL-6 is involved in the development of ischemia-reperfusion injury, its deficiency having beneficial effects on infarct size, independently of IL-1β, TNFα levels, or neutrophil influx [127]. A meta-analysis conducted by Yang et al. has shown that the severity of acute coronary syndromes significantly correlates with IL-6 levels [128]. High IL-6 levels are associated with a decreased ejection fraction and a larger infarct size at the 4-month follow-up visit after STEMI [129]. Furthermore, in the ACS setting, IL-6 outperformed the accuracy of CRP in predicting long-term cardiovascular death [130]. The molecular mechanisms by which IL-6 causes cardiomyocytes’ apoptosis and remodeling-related ischemia-reperfusion deserve a deeper insight. Nguyen et al. report high levels of IL-6 being a predictor of cardiovascular complications in COVID-19 patients [67] (Table 1). Persistently high levels of IL-6 during the recovery phase of the infection may contribute to impaired left ventricular function. Moreover, high levels of cTn suggesting ongoing myocardial injury were detected in these patients [131].

Interleukin 10 (IL-10) is an anti-inflammatory cytokine synthesized by various immune cells, including macrophages and lymphocytes. It exerts its anti-inflammatory effects by activating T lymphocytes and by directly inhibiting the innate immune-related functions of dendritic cells and macrophages [138]. In patients with SARS-CoV-2 infection, an early, substantial rise of IL-10 was observed, along with IL-6 as a disease severity predictor. Increased levels of IL-10 may be a consequence of the attempt to reduce the inflammatory response, thus preventing the cytokine storm [139]. 

Nevertheless, simultaneously high levels of IL-10 and pro-inflammatory cytokines, as well as the correlation with disease severity, may be explained either by the inability of the cytokine to reduce inflammation or by a dysregulated function [140,141,142]. Controversial evidence supporting the potential pro-inflammatory role of IL-10 outside of COVID-19 indicates that hyperactivation of CD8+ T cells and their functional exhaustion of this biomarker may contribute to the interplay of the cytokine storm [143]. The protective effect of IL-10 has been demonstrated in animal studies, with low levels being correlated with extensive atherosclerotic lesions, doubled by a higher risk of plaque rupture [144]. IL-10 wields an anti-atherogenic role from the initiation of plaque formation by modulating lipid metabolism in macrophages [119]. However, in human subjects, IL-10 levels are highly variable, being elevated, decreased, or stationary in patients with stable coronary artery disease or unstable angina, as compared to controls [145,146,147]. 

Furthermore, controversies persist also concerning IL-10′s prognosis value; in patients with ACS, high IL-10 serum levels may be related to either an improved or worsened outcome [132,148,149]. Administration of recombinant IL-10 has been proposed by some authors for the therapeutic management of ARDS in COVID-19, but clinical evidence has shown that it might be detrimental as it promotes T cell exhaustion [34,141]. Further clinical studies are required in order to elucidate all of its functions and outline the complete profile of IL-10.

### 3.3. Tumor Necrosis Factor Alfa (TNF-α)

TNF-α is a pro-inflammatory cytokine promoting the SARS-CoV-2 interaction with ACE 2 and taking part in cytokine releasing syndrome (CRS) together with IL-1, IL-6, and IL-10. It is an important cytokine of the innate immune system, inhibiting viral replication and carcinogenesis [101]. Song et al. reported that plasma levels of TNF-α, IL-6, and CRP are positively correlated with acute cardiac injury [69]. 

### 3.4. D-Dimers

Assessing the inflammatory status in the evaluation of coagulant status is important since ubiquitous coagulation abnormalities are found in critically-ill patients. D-dimer levels have shown the same pattern as cTn in patients with severe forms of the disease, increasing progressively as patients’ clinical status worsened [150]. The high affinity of SARS-CoV-2 for endothelial cells might induce apoptosis of vascular cells with the enhanced release of prothrombotic molecules, thereby triggering not only a local but also a systemic coagulopathy that may affect the pulmonary vessels as well as coronary arteries, with the subsequent microthrombi formation and extensive cardiopulmonary injury [38,151]. Accordingly, D-dimer serum levels represent an accurate predictor for mortality in COVID-19 pneumonia as it was demonstrated that these molecules may also reflect certain phenomena that are associated with illness severity, such as extravascular fibrinolysis, interstitial pulmonary edema, and lung injury in COVID-19 patients without venous thromboembolism [152]. Concerning optimal cut-off values indicative of a poor prognosis, literature data reported figures ranging from 1.5 to 2.1 mg/L [153,154].

### 3.5. C-Reactive Protein (CRP)

A surge in serum levels of CRP is a consistent finding in infectious pathology, with COVID-19 as no exception. CRP is a non-specific, liver-synthesized, acute-phase protein that is related to essential inflammatory and immune processes, such as apoptosis, phagocytosis, NO release, the complement pathway, and regulates cytokine production, such as IL-6 or TNF-α [155]. Moreover, extensive literature data highlighted that elevated CRP serum levels are independent predictors of mortality not only in COVID-19 [71] but also in patients with cardiovascular pathology, particularly related to atherothrombosis, thus outlining CRP as a dual, cardio-inflammatory biomarker, and a useful prognosis tool in the pandemic, given its routine, widespread measurement in clinical practice [156,157].

The use of high-sensitivity assays (hs-CRP) enhances its ability to identify severe forms of the disease in the early stages, as several data reported that especially high CRP levels occur in severe cases compared to non-severe ones, and higher concentrations being reported in COVID-19 patients who developed shock, ARDS, or acute cardiac injury [158,159] Interestingly, a very recent study by Lionte et al. highlighted that elevated hs-CRP was associated with a poor prognosis in non-critically ill patients with COVID-19, thus further supporting the hypothesis of a sustained, both pulmonary and systemic impact, of SARS-CoV-2 related inflammation [160].

Another potentially useful prognosis tool is represented by the dynamic assessment of hs-CRP. The CRP velocity refers to the variation of this biomarker’s serum levels after seriated measurements; recent results already demonstrate the correlation between increasing hs-CRP and a poorer LVEF following an acute myocardial infarction [161], the same pattern observed in the case of COVID-19’s outcome, as reported in an extensive meta-analysis [162].

CRP has also been used as a therapeutic target in a small number of patients with respiratory failure and severe pulmonary infiltrates. After selective apheresis of CRP, an improvement in the radiological appearance was observed, suggesting that performing this technique immediately after admission could significantly reduce pulmonary injuries [163]. Two studies have established that patients with CRP values above 41.8 mg/L and 41.4 mg/L, respectively, have a higher risk of progressing to a severe form of the disease [164,165]. In patients already diagnosed with a severe form of COVID-19, our research team recently identified a high-risk CRP concentration cut-off; a value above 23 mg/L is associated with a negative vital prognosis [70].

### 3.6. N-Terminal Pro-Brain Natriuretic Peptide (NT-proBNP) 

NT-proBNP is a “classical” biomarker in HF, being correlated with both elevated left ventricular filling pressures and systolic dysfunction [166]. In SARS-CoV-2 infection, the dynamic changes of NT-proBNP, CRP, and cTn concentrations are basically related to the extent of the inflammatory response, tissue injury, and functional impairment. Guo et al. reported higher levels of natriuretic peptides in severe forms of COVID-19 pneumonia, especially in deceased patients [26], while De Falco et al. showed that the prognosis value of NT-proBNP is similar to cTn, specifically for mortality risk stratification [136].

However, it is difficult to strictly correlate the presence of COVID-19 with the cardiovascular lesions evolving with increased biomarkers. Multiple studies have shown that elevated serum levels of cTn or NT-proBNP are similarly associated with a poor prognosis in a plethora of potentially COVID-19-related pathologies, such as pneumonia, septic shock, or acute respiratory distress syndrome [88,167,168]. For example, patients with pneumonia who had elevated concentrations of NT-proBNP were more prone to be admitted to ICU wards, regardless of the presence of cardiac dysfunction. There is growing evidence supporting the hypothesis of hypoxia-induced vasoconstriction that ultimately leads to pulmonary hypertension, which in turn may increase parietal RV stress in patients with extensive pulmonary lesions, thereby causing an increased NT-proBNP release from cardiomyocytes, a very plausible mechanism in the context of COVID-19 [168,169,170]. An additional inducer of myocardial parietal stress may be the widespread use of vasopressors in patients with septic shock, while acute kidney injury in severely ill patients may alter the clearance of NT-proBNP, leading to a false, non-cardiac increase in these biomarkers [88,170]. Other COVID-19-related causes for an increased NT-proBNP include oxidative stress, arrhythmias due to disease, per se, or due to its specific medications, and microvascular ischemia due to a demand-supply mismatch [82]. The importance of this mosaic characterized by high sensitivity and low specificity resides in the persistently strong correlation between NT-proBNP and mortality rates in severe COVID-19 patients, even after the exclusion of cases presenting HF [171].

Under these pandemic circumstances, it is essential to investigate a biomarker capable of detecting subclinical myocardial injury at an early stage in COVID-19 patients. For example, the soluble isoform of ST2 (sST2) is a very promising cardiac biomarker, not only for initial assessment but also for the long-term prognosis of patients with HF and COVID-19.

### 3.7. ST2

ST2 is a member of the extensive interleukin 1 (IL-1)/Toll-like receptor (TLR) family, more commonly known as interleukin 1 receptor-like 1 (IL1RL-1). Different cardiovascular effects of the ST2 molecule have been observed, depending on the isoform under which it is expressed: a transmembrane receptor form (ST2L) and a circulating, soluble, receptor form (sST2) that can be assessed in serum using various ELISA-based kits [88,137,168]. The ST2L also represents the receptor for IL-33, exercising cardioprotective actions through binding, such as inhibiting myocardial fibrosis, limiting ventricular hypertrophy, and preventing early apoptosis. On the other hand, the sequestration of IL-33 in the bloodstream by the decoy sST2 isoform prevents the occurrence of these positive effects, also representing the substrate of its high serum levels in patients with HF or with other myocardial injuries.

The diagnostic and prognostic role of ST2 in cardiovascular pathology has been validated by numerous studies, especially in patients with acute HF, as it is considered a reliable marker of fibrosis, ventricular wall strain, and increased filling pressures, also accurately predicting re-hospitalizations and fatal events [168,172,173,174].

The potential role of ST2 as a dual cardio-inflammatory biomarker was highlighted by some authors that observed abnormally elevated levels of sST2 in patients who tested positive for SARS-CoV-2 infection without presenting cardiovascular comorbidities [175]. Very interestingly, there is additional evidence reporting even a superior concentration of ST2 among COVID-19 patients compared to chronic, clinically stable HF [176,177].

The IL-33/ST2 axis also represents an essential pathway for pulmonary lesion and lung disease progression. IL-33 is an alarmin-type cytokine whose gene is overexpressed under stressful local conditions, such as an infection leading to alveolar injury or necrosis. As opposed to the positive effect at the myocardial level, the interaction between IL-33 and ST2L promotes inflammation by inducing the TGF β-mediated differentiation of T-cells and the increased secretion and release of pro-inflammatory cytokines or chemokines (e.g., IL-6, IL-1β, TNFα, IL-8, IL-4, IL-5, and IL-13), with a subsequent persistence and even amplification of inflammation’s deleterious consequences. Therefore, the detection of high serum levels of sST2, the “decoy” receptor isoform, may suggest a regulatory mechanism for limiting inflammation and for preventing further inflammatory cell inflow and consecutive tissue damage [178]. Moreover, the IL-33/ST2 axis plays an important role in pulmonary fibrosis, as shown in two experimental models in which mice treated with a profibrotic substance exhibited a linear increase in IL-33 expression, an aspect explained by the alleged activation of fibroblasts by inflammatory cytokines triggered by the interaction between IL-33 and ST2L [179,180].

Based on these findings, it is reasonable to assume that COVID-19 may drastically activate the IL-33/ST2 system in a way similar to that observed for other inflammatory markers, directly affecting patients’ outcomes via sST2′s negative cardiovascular impact, such as pro-hypertrophic and pro-apoptotic effects [178,181].

Cytokine releasing syndrome (CRS) refers to a severely unregulated immune response to infection. The hallmark of CRS is represented by the proliferation and activation of lymphocytes and macrophages, followed by a significantly increased release of cytokines [182]. As previously mentioned, the cytokine superfamily comprises interleukins, chemokines, tumor necrosis factors, interferons, transforming growth factors, and colony-stimulating factors [96]. The upstream cytokine IL-1β induces IL-6 synthesis which increases vascular permeability, causing myocardial interstitial edema [183] and decreased myocardial contractility [184]. Cardiotoxicity, in the setting of CRS, is clinically expressed by left ventricular dysfunction, hypotension, and even cardiogenic shock [185]. Elevated levels of IL-6 in hospitalized patients with COVID-19 are directly correlated with high levels of cTn. These findings support the potential role of IL-6 in SARS-CoV-2-related acute cardiac injury during cytokine storms [34]. 

The COVID-19 pandemic turned the spotlight on the routine determination of cardiac biomarkers in SARS-CoV-2 infected patients. Multiple studies already confirmed that cTn, NT-proBNP, ST2, IL-6, IL-10, IL-1, and TNF-α represent relevant biomarkers that are useful for risk stratification in patients with acute cardiac injury due to COVID-19 pneumonia [88]. Moreover, given the well-established relationship between inflammation and atherosclerosis, a reasonable approach would consist of an assessment of subclinical markers of atherosclerosis, such as carotid intima-media thickness, pulse wave velocity, and ankle-brachial index, which are readily determined and well correlated with multiple cardiovascular risk scores, such as SCORE or Framingham [186]. Keeping in mind the socioeconomic challenges associated with the pandemic and the increased prevalence of cardiovascular comorbidities among admitted patients, we consider that judicious use of all these available diagnostic and prognostic biomarkers may facilitate the identification of individuals with a high-risk profile, thus allowing a prompt and adequate therapeutic intervention, as the limitation of myocardial injury during SARS-CoV-2 infection is of paramount importance for both short and long-term prognosis [134,187].

## 4. Novel Therapeutic Strategies: The Frontline against Inflammation

Studies investigating the efficacy of IL-1 and IL-6 inhibition in lowering mortality rates in patients with SARS-CoV-2 infection have controversial results at the moment, but several trials are currently underway [188]. Biologic response modifiers, also known as immunomodulators, are being used as targeted drug therapy in autoimmune diseases and cancers [189]. The protective myocardial role of immunomodulatory therapies is reported for Tocilizumab and Sarilumab monoclonal antibodies, being agents that block both the soluble and the membrane IL-6 receptors. Additionally, these molecules have been shown to play a significant role in reducing systemic inflammation. Despite its negative effect on the lipid profile, Tocilizumab administration in patients with high cardiovascular risk has led to outstanding improvement in endothelial function [190]. Elevated levels of proinflammatory biomarkers during COVID-19 increase the risk of acute myocardial infarction, but also have an impact on its size [191]. The effectiveness of Tocilizumab in limiting the infarction area and improving the prognosis was demonstrated in a randomized, double-blind, placebo-controlled trial evaluating the myocardial rescue index. Broch et al. have found that the myocardial rescue index measured by magnetic resonance imaging was significantly higher after Tocilizumab administration. Less extensive microvascular obstruction and a significant reduction in CRP levels have also been observed in patients receiving the IL-6 receptor blocker [56]. Tocilizumab was not effective for significantly reducing levels of troponin and NT-proBNP in patients hospitalized with COVID-19. Moreover, an IL-6 blockade was not notably associated with reduced mortality rates in patients admitted for SARS-CoV-2 infection, complications with ARDS, and exacerbated inflammation [188,192].

Different regimens of Anakinra, a recombinant IL-1 receptor antagonist, administered early in patients with moderate immune hyperactivation during COVID-19 pneumonia were associated with reduced mortality risk and a good safety profile [192,193,194]. Conversely, in a randomized double-blinded controlled-placebo trial, Canakinumab, a fully human monoclonal antibody neutralizing IL-1β, did not significantly increase survivability [195]. The Three C Study, an ongoing randomized controlled trial (NCT04365153) is investigating the role of Canakinumab in improving outcomes in patients with SARS-CoV2-associated myocardial injury and hyperinflammatory status. The dynamics of cardiac enzymes and cardiac function should be further investigated in patients with acute cardiac injury or even ACS and COVID-19 receiving monoclonal antibodies. Promising evidence regarding the effect of immunomodulatory strategies in the setting of ACS is currently emerging, as Canakinumab demonstrated a reduction in mortality and hospitalization for HF in patients with a personal history of myocardial infarction and elevated CRP [104]. Moreover, the use of Anakinra in patients with STEMI decreased all-cause mortality and incidence of newly diagnosed HF without affecting the left ventricular ejection fraction compared to placebo [10]. These data suggest that drugs targeting cytokines may be effective in limiting myocardial damage in SARS-CoV-2 infection as well.

Taken together, IL-6 inhibition yields no benefit in COVID-19 pneumonia progression, while an IL-1 blockade has shown only moderate benefit. A more robust clinical positive impact exerted by IL-1 might be its upstream position in the inflammatory cascade; compared to IL-6, a successful IL-1 inhibition potentially leads to the downstream suppression of IL-6 and other inflammatory biomarkers [104,192]. A current matter of debate and future investigations is if an IL-6 and IL-1 blockade truly offers advantages over the standard management in COVID-19, particularly if associated with acute cardiac injury. Additional studies regarding the benefit, safety, and regimens of immunotherapy in preventing and treating the cardiovascular complications of COVID-19 are needed [97,180,185,196].

## 5. Conclusions

Currently, it is generally accepted that the SARS-CoV-2 infection is associated with an increased prevalence of acute cardiac injury, regardless of potential underlying cardiac causes. The abnormal immune response, endothelial dysfunction, and O_2_ supply-demand imbalance are the essential pathophysiological mechanisms contributing to the disease severity in COVID-19, while also being common features in acute cardiac injury. Direct myocardial injury, along with a plethora of indirect mechanisms, represents the substrate of myocardial damage in SARS-CoV-2-related acute cardiac injury pathogenesis. The occurrence of myocardial injury portends a severe course of the disease and is an independent predictor of mortality, therefore an early diagnosis combined with prompt therapeutic management represents the cornerstones for the short-term prognosis of infected patients. In this context, it is essential to make use of the full array of diagnostic tools, highlighting the growing role of cardio-inflammatory biomarkers. An effective assessment of inflammatory status may act as a bridge toward the identification of new, effective, therapeutic strategies that address both COVID-19 and the eventual cardiovascular complications.

## Figures and Tables

**Table 1:** Biomarkers suggesting acute cardiac injury in COVID-19.

IL-1β	High levels are associated with a hypercoagulable state and thrombotic events, worsening the prognosis [68]
IL-6	Elevated levels correlate with the occurrence of major adverse cardiac events and/or mortality risk [67]
IL-10	Increases ACE 2 expression and may promote endothelial cell infection and vasculitis [132]
TNF-α	Positively correlated with acute cardiac injury, disease severity, and high mortality rates in ICU patients [69]
IFN-γ	Elevated levels detected in cytokine storm cause multiple organ dysfunction [133]
CRP	Independent predictor of very high cardiac troponin levels, associated with high rates of all-cause mortality [134]
Troponin	High levels indicate a risk of death 8 to 21 times higher [10,11]
D-dimers	COVID-19-associated coagulopathy, biologically expressed through increased D-dimer and prolonged PT is a predictor of myocardial injury and poor prognosis [135]
NT-proBNP	Prognostic value similar to troponin in survival rates [136]
ST2	Superior prognostic value compared to NT-proBNP [88,137]
